# The Effectiveness of Multi-Component Interventions on the Positive and Negative Aspects of Well-Being among Informal Caregivers of People with Dementia: A Systematic Review and Meta-Analysis

**DOI:** 10.3390/ijerph19126973

**Published:** 2022-06-07

**Authors:** Jinjie He, Jing Wang, Hongmei Zhong, Chengguo Guan

**Affiliations:** 1 Faculty of Nursing, Health Science Center, Xi’an Jiaotong University, #76 Yanta West Road, Xi’an 710061, China; hjj521209@stu.xjtu.edu.cn (J.H.); novogcg@stu.xjtu.edu.cn (C.G.); 2 Department of Nursing, School of Medicine, Shihezi University, Shihezi 832002, China; 15899298234@163.com

**Keywords:** Alzheimer disease, dementia, informal caregivers, mental health, meta-analysis, systematic review

## Abstract

The present review aims to examine whether multi-component interventions for informal caregivers of people with dementia are effective on positive and negative aspects of caregiver well-being. Eleven databases were searched from inception to 8 March 2021. Only randomized controlled trials reporting the effectiveness of multi-component intervention on positive and negative aspects of caregiver well-being were eligible. Endnote X7 (Thomson ResearchSoft, Stanford, CA, USA) was used for study selection and version 5.1.0 of Cochrane Collaboration’s tool (Cochrane, London, UK) was applied for quality assessment. Review Manager (Revman) Version 5.3 (Cochrane, London, UK) was used for the meta-analysis, and if statistical synthesis was inappropriate, only narrative analysis was performed. A total of 31 RCTs with 3939 participants were included. Meta-analyses showed small to moderate effects on subjective well-being, depression, and burden of caregivers, and a moderate to high effect on caregiver anxiety. Due to insufficient data and vast heterogeneity, meta-analysis was not performed for other outcomes, such as resilience, competence, and empathy. This review suggests that individualized multi-component interventions for caregivers may be one of the ways to promote their well-being. Further research is needed to explore the impact of rigorously designed and personalized multi-component interventions on informal caregivers, especially on more positive indicators, as well as its long-term effects and sustainability.

## 1. Introduction

Dementia is a leading cause of disability among people aged 65 years and over [1]. It is estimated that there are approximately 50 million people living with dementia worldwide, and the number is forecasted to reach 82 million by 2030 and 152 million by 2050 [2]. As the life expectancy of the world’s population increases, the number of people with dementia continues to grow [3]. Up to 94% of people with dementia are cared for by informal caregivers, who have become the backbone of informal care [4]. Caring for people with dementia puts a significant impact on the physical condition, mental health, well-being, and social relationships of informal caregivers as evidenced by the high-level burden and high prevalence of mental health problems among caregivers, which contributes to the poor quality of care and quality of life of caregivers and those they care for [5]. Effective interventions that can support informal caregivers to manage negative emotions and enhance well-being are therefore required.

Well-being is usually described as a theoretical construct, which includes emotional (affects/feelings), psychological (positive functioning), social (relations with others), and spiritual (sense of purpose in life) aspects [6,7], and it reflects not only the relative absence of some undesirable outcomes but also the presence of positive aspects [8]. More and more evidence shows that both positive and negative feelings exist in the care process of informal caregivers [9,10]. Previous studies have focused more on the impact of interventions on negative outcomes. However, caregivers pay too much attention to negative emotions, which may result in a pessimistic spiritual outlook and secondary harm, in ignoring self-affirmation of successful performance [11]. The more negative thoughts the caregiver has, the likelier they are to experience a stronger sense of overload. Conversely, when caregivers focus on the positive aspects of care, it seems to improve their mood [12]. Positive feelings help to improve the intimate relationship between the caregiver and the people with dementia and to promote the caregiver to actively respond to the problems faced in the care process [13].

Multi-component interventions appear to have a good potential for improving caregiver well-being outcomes [14]. It means any intervention containing elements of at least two of the categories. These categories include, but are not limited to, psychoeducational interventions, psychotherapy, skills training, professional or peer support, respite care, case management, exercise, attendance at a memory clinic, meditation/mindfulness, and so on [15]. Multi-component interventions can be delivered in multiple methods (i.e., face-to-face, telephone, or online) and are conducted by the trained health professional in dyadic approaches or by the caregiver alone. These well-designed and clearly structured multi-component interventions for caregivers of people with dementia are intended to improve their positive aspects of caregiving, reduce their burden and depressive symptoms [16], and even delay the institutionalization of people with dementia [17] by individualized content for caregiver demands. Importantly, multi-component interventions have been widely used in informal caregivers of disabled elderly people [18], and similar results have been found in caregivers of people with dementia.

The recent findings regarding the effects of multi-component interventions on negative aspects of caregiver well-being of informal caregivers are inconsistent. For example, the results of some randomized controlled trials (RCTs) have indicated that the depression [19,20,21], burden [19], or anxiety [21] of caregivers was significantly ameliorated in the multi-component intervention group compared with the control group. On the contrary, some studies have shown that there were no significant differences in the depression [22,23,24], burden [20,22], or anxiety [20,24] of caregivers between the multi-component groups and control groups. Currently, there are several systematic reviews of multi-component interventions for informal caregivers of people with dementia. However, in these previous systematic reviews, the authors focused on the optimal way to combine multiple components [25], the impact on a single positive outcome such as competence in caregiving [26], or the impact on negative outcomes [27]. There is a gap in systematic evaluations exploring the effects of multicomponent interventions on both positive and negative aspects of caregiver well-being. Although the effect of multicomponent interventions on caregivers’ subjective well-being was explored in a recent systematic evaluation of a nonpharmacological intervention, no statistical significance was found. In addition, it included only a small amount of literature published in English and German and did not explore the effects on other outcomes such as self-efficacy, empathy, and resilience [28]. Therefore, it is necessary to conduct a thorough systematic review to explore the impact of multi-component interventions for dementia caregivers on both positive and negative aspects of well-being.

This review focused on RCTs investigating the effectiveness of multi-component interventions on positive and negative aspects of well-being in informal caregivers of people with dementia and compared the intervention effects of different delivery methods, including caregivers only or dyadic interventions. Findings from this review will provide evidence of effectiveness of multi-component interventions on caregiver well-being and enable health professionals to become aware of the positive aspects of caregiver well-being, which will inform the development of effective multi-component strategies and dementia care services for informal caregivers.

## 2. Materials and Methods

### 2.1. Design

A systematic review and meta-analysis were performed with reference to the Cochrane Handbook of Intervention Studies [29], and this paper was developed following the PRISMA statement (Table S1).

### 2.2. Search Methods

We searched the ALOIS, Medline, Embase, LILACS, PsycINFO, CINAHL, Web of Science English databases, and Wanfang, CBM, VIP, and CNKI Chinese databases for all articles published prior to July 2020. The retrieval of the databases above was updated on 8 March 2021, and a combination of the following search terms and Medical Subject Heading terms were used: ‘Alzheimer’; ‘dementia’; ‘informal caregiver’; ‘family’; ‘relative’; ‘spouse’; ‘multi-component’; ‘combinate’; ‘comprehensive’; ‘random’; and ‘RCT’. The full electronic strategy for the databases is listed in Table S2.

### 2.3. Inclusion and Exclusion Criteria

Studies were included if they had adult informal caregivers who provided unpaid care for people with any type of dementia in the home settings. Second, studies were RCTs in which informal caregivers were randomly assigned to a multi-component intervention group or to a control condition (e.g., usual care, wait-list control, alternative intervention, single-component intervention). Third, multi-component interventions, which means any intervention containing elements of at least two of the different intervention categories had to be included [26]. As defined in guidelines published by the National Institute for Health and Care Excellence in 2018, these categories include psychoeducational interventions, psychotherapy, skills training, professional or peer support, respite care, case management, exercise, attendance at a memory clinic, meditation/mindfulness, and so on [15]. Fourth, the intervention routes included face-to-face, telephone, and online approaches, and the form of the intervention included both individuals and groups. Both interventions that recruited caregivers only and dyadic interventions with informal caregivers as the main intervention target were included. Fifth, they had to report caregiver outcomes in any of the following categories: subjective well-being, relationship satisfaction, resilience, self-efficacy, empathy, competence, burden, anxiety, depression, and stress. Lastly, they were full-text articles published in English or Chinese.

Studied were excluded if (1) participants were diagnosed with mild cognitive impairment, or both dementia and mild cognitive impairment, but the data could not be distinguished; if (2) interventions containing multiple forms from the same category (e.g., an intervention only including both group and individual psychoeducation intervention); and if (3) it is a letter, commentary, case report, conference abstract, literature review, systematic review, or meta-analysis.

### 2.4. Types of Outcome Measures

Subjective well-being was the primary outcome of this study. Subjective well-being is one of the positive indicators of intrinsic aspects of well-being, which is usually described as a positive aspect of well-being, so that individuals can fully interact with others, deal with life pressure and realize their own abilities [8]. Based on the literature review and the concept map of well-being proposed by previous studies [8,30], the secondary outcomes included self-efficacy, depression, anxiety, stress, burden, relationship satisfaction, resilience, empathy, and competence.

### 2.5. Study Selection

First, one reviewer merged references from different databases in Endnote X7 and deleted duplicates. Second, two reviewers independently screened titles and abstracts to select articles for full-text review. Third, two reviewers conducted a full-text review to determine which studies met inclusion criteria. Finally, we resolved disagreements through discussion or a third reviewer.

### 2.6. Quality Appraisal

Two reviewers independently completed the assessment of the risk of bias and any disagreements were discussed and resolved with the third reviewer in regular team meetings. The Cochrane Collaboration’s tool (version 5.1.0) was applied for quality appraisal [31], including seven domains of bias: sequence generation, allocation concealment, blinding of participants and personnel, blinding of outcome assessment, incomplete outcome data, selective outcome reporting, and ‘other issues’. Each domain in the tool was rated as “low risk”, “high risk” or “unclear risk”. The Cochrane Handbook of Intervention Studies suggests that if multiple domains in a study are judged to be “Unclear” in a way that substantially lowers confidence in the result, the study is considered to be at high risk of bias. In addition, assessing the risk of bias due to missing results is an essential component of a Cochrane Review, so studies that were evaluated as low risk of bias in the domain of “Incomplete outcome data“ were included in this systematic review [29]. Three articles were excluded during the quality appraisal due to many unclear risks of bias and the domain of “Incomplete outcome data” was unclear [32,33,34].

### 2.7. Data Extraction

The family name of the first author, year of publication, country, participants, mean age, the proportion of females, condition of intervention and control group, sample size, outcomes, and follow-up time points were extracted in a standardized data extraction form and were checked for extraction accuracy for analysis in Revman Version 5.3 (Cochrane, London, UK).

### 2.8. Synthesis

All statistical analyses were conducted using Revman Version 5.3 when the mean and standard deviation of the outcome data were reported or calculated. Otherwise, studies were described narratively. Considering that the outcomes of the included studies were measured using different scales and that many combinations of multi-component interventions were included for review, the standardized mean difference (SMD) and 95% confidence interval (95% CI) between the scores of the two groups immediately after the intervention were calculated to estimate the overall intervention effect [29].

Cochran’s Q test and I^2^ statistic were used to estimate statistical heterogeneity. A *p* value of Cochran’s Q test < 0.1 indicated heterogeneity among included studies. I^2^ heterogeneity test was <40%, 40–75%, >75%, indicating low, moderate, and high consistency of the included studies, respectively. A fixed effect model was used in this review if there was no significant heterogeneity (*p >* 0.1, I^2^ < 40%). When included articles showed moderate heterogeneity (*p <* 0.1, 40% < I^2^ < 75%), we firstly checked and confirmed that the data entered into the software were accurate and then a random effect model was used to combine effect sizes [29]. If heterogeneity was high (*p <* 0.1 and I^2^ > 75%), the sources of heterogeneity were explored in terms of clinical heterogeneity and methodological heterogeneity. Subgroup analyses based on the type of participant and delivery method were performed according to the protocol design to examine the source of heterogeneity. When heterogeneity was too pronounced and unresolvable, meta-analysis was inappropriate and only narrative analysis was performed. The sensitivity analysis was performed to test the robustness of the meta-analysis results. When the number of articles included for an outcome was ≥5, publication bias of the articles were assessed visually using funnel plots and statistically by Egger’s test.

## 3. Results

### 3.1. Study Selection

**Figure 1:**
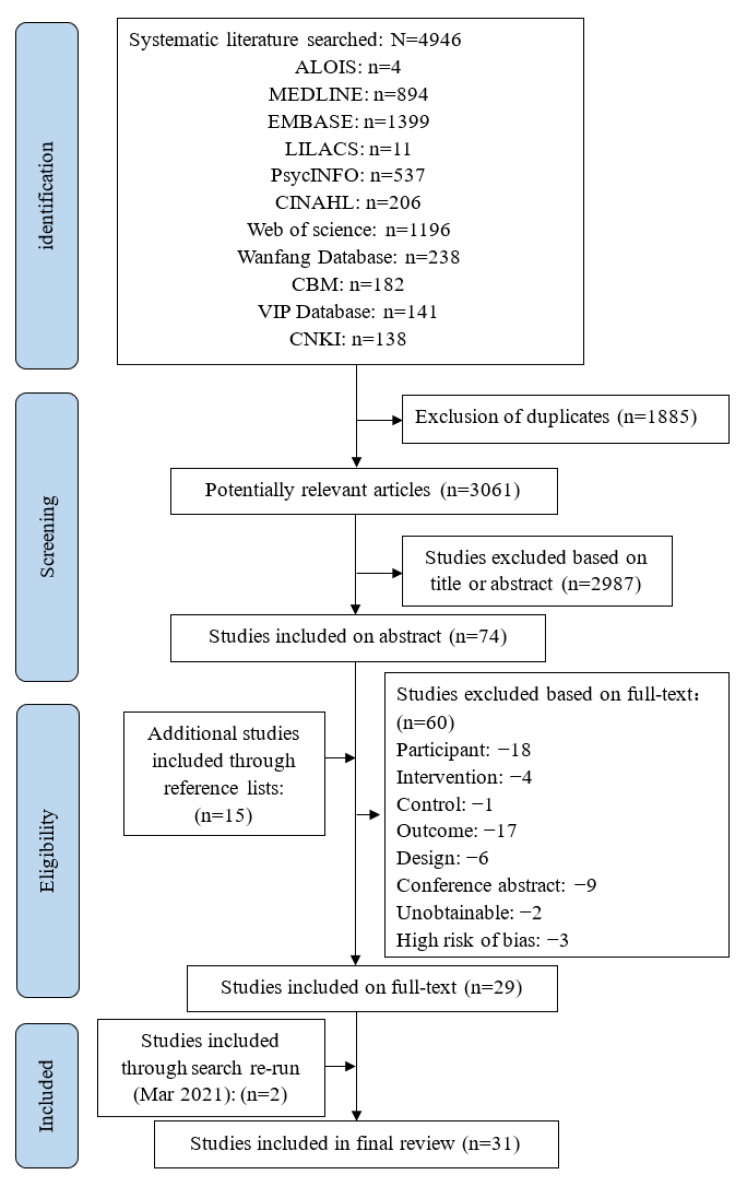
Flowchart for studies selection.

The study selection process is presented in Figure 1. A total of 4946 potentially relevant citations were identified from 11 databases and imported to Endnote X7. Totally 1885 duplicate articles were excluded in the first step. After screening the titles and abstracts, 2987 articles were excluded. In all 60 articles were excluded after quality appraisal and reading the full text. Fifteen relevant articles were included by manual exploration of the reference lists of the included studies. We searched the databases again on 8 March 2021, and updated two studies. Finally, 31 studies published between 2000 and 2020 were included, 24 in English and the rest in Chinese.

### 3.2. Study Characteristics

In total, the 31 studies included 3939 informal caregivers, of whom 2080 were allocated to the intervention groups and 1859 to the control groups. Studies typically recruited more women (50.6–93.1%) than men, and the average age of caregivers ranged from 48.0 to 77.3 years. Most studies involved caregivers only, and 10 articles included both people with dementia and their caregivers (dyadic) [18,19,23,35,36,37,38,39,40,41]. Of the included studies, 15 studies (48.4%) were conducted or assisted by nurses, 6 studies were conducted by psychotherapists, and the remaining 10 studies were led by trained research team members or social workers. Regarding the intervention delivery methods, 21 studies conducted face-to-face interventions, three studies focused on telephone interventions, and seven studies focused on online methods. The number of interventions implemented across the included literature ranged from 4 to 16 and were usually held once a week or every two weeks, with duration ranging from 20 to 240 min. In all included studies, the outcomes were measured before and after the intervention. However, the duration of the intervention ranged from 1 to 48 weeks. Table S3 summarizes the characteristics of the included studies.

### 3.3. Risk of Bias

The method of random assignment sequence generation was described in 83.9% of the included studies, but the exact implementation of their allocation concealment was unclear in more than half of the studies (54.8%). Due to the nature of the intervention, only 32.3% of the studies were able to blind participants and personnel, and 54.8% of the studies were able to blind outcome assessors. All the studies reported the number and reason for dropouts. Up to 87.1% of the studies had a low risk of reporting bias, and 11 articles (35.5%) had either published protocols or were registered on clinical trial registries. In addition, one study [36] may have led to other biases due to the particular clinical setting (Figure S1).

### 3.4. Impact on the Positive Aspects of Caregiver Well-Being

The outcomes in this review included both positive and negative aspects of caregiver well-being. The following are outcomes for the positive aspects of well-being, including caregiver’s subjective well-being, self-efficacy, relationship satisfaction, resilience, empathy, and competence.

#### 3.4.1. Impact on the Caregiver’s Subjective Well-Being

**Figure 2:**
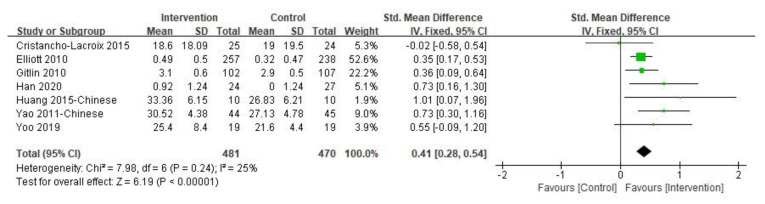
Effect of multi-component interventions on informal caregiver’s subjective well-being (post-intervention).

The caregiver’s subjective well-being was the primary outcome, the results of which are shown in Figure 2. The duration of the intervention varied from 2 h to 12 months. In the included studies, subjective well-being was measured by different instruments, such as two studies used the Positive Aspects of Caregiving and others used the Nottingham Health Profile, a mood improvement questionnaire, the Perceived Change Index, the Positive Affect and Negative Affect Schedule, and the Care-Related Quality of Life Instrument-Visual Analogue Scale tool, respectively. The overall pooled SMD for the seven studies was significant [SMD = 0.41, 95% CI (0.28, 0.54), *p <* 0.001, I^2^ = 25%], with effect size estimated as small to moderate. Sensitivity analysis was performed by omitting one study in each round and the results did not change. Only two studies [36,42] investigated the subjective well-being of informal caregivers at three-month follow-up and no significant difference was found (*p >* 0.05). However, one study reported that the intervention group’s average subjective well-being score increased during the follow-up compared with the post-intervention results, while the control group’s average score decreased [42].

#### 3.4.2. Impact on the Caregiver’s Self-Efficacy

**Figure 3:**
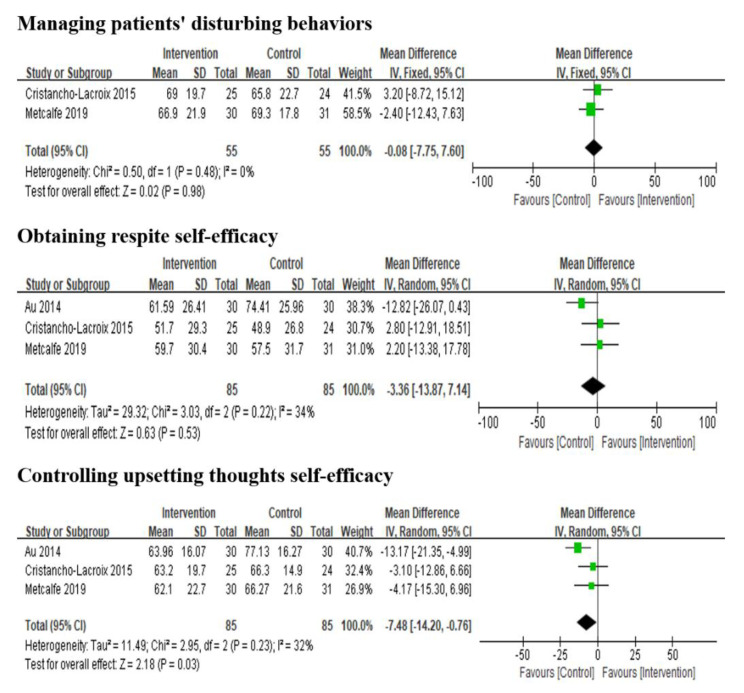
Effect of multi-component interventions on informal caregiver’s self-efficacy (post-intervention).

Five studies reported the effects of multi-component interventions on caregiver self-efficacy analysis. It is important to note that Duggleby et al. [43] and Possin et al. [19] used the General Self-efficacy Scale (GSES) and the Care Ecosystem Caregiver Self-efficacy Scale to assess participants’ perceived self-efficacy, respectively. Both studies found no significant improvement in self-efficacy. The other three studies [22,42,44] used the Revised Scale for Caregiving Self-efficacy (RSCSE) to measure self-efficacy in three dimensions: managing patients’ disturbing behaviors, obtaining respite and controlling upsetting thoughts. The results reported that multi-component interventions had no significant impact on the three dimensions of caregiver self-efficacy (Figure 3).

#### 3.4.3. Impact on Other Positive Outcomes

Relationship satisfaction was reported in two studies [SMD = 3.48, 95% CI (0.95, 6.02), *p* = 0.007, I^2^ = 0%] [42,44], and one study with a weight of 96% had statistically significant and reported higher scores in the multi-component interventions group [45].

Two studies reported the empathy and competence of caregivers, and a meta-analysis was not performed due to insufficient data [39,45]. Except for the results of Hattink et al. [45], which showed that multi-component intervention may improve caregiver empathy, other results were not statistically significant. Only Kor et al. [20] assessed the resilience of caregivers using the Brief Resilience Scale. The 10-week intervention and the third-month follow-up results showed that multi-component interventions could not improve the resilience of caregivers (*p >* 0.05). However, the mean score of resilience in the intervention group increased during the follow-up period, while the mean score of the control group decreased.

### 3.5. Impact on the Negative Aspects of Caregiver Well-Being

The following are outcomes for the negative aspects of well-being, including caregiver’s depression, burden, anxiety, and stress.

#### 3.5.1. Impact on the Caregiver’s Depression

**Figure 4:**
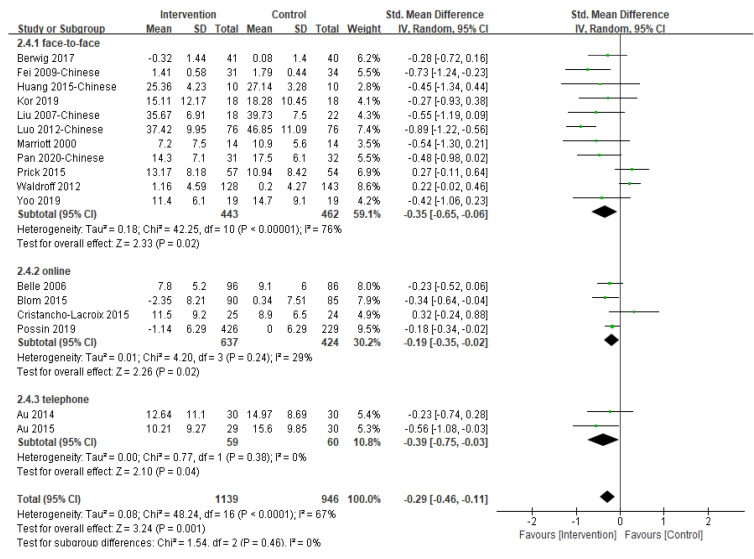
Effect of multi-component interventions on informal caregiver’s depression (post-intervention).

Eighteen of the included 31 studies reported the post-intervention depression symptoms of caregivers and 17 provided sufficient data for inclusion in meta-analysis (Figure 4). Of the 17 studies included in the analysis, six studies used the Centre for Epidemiologic Studies Depression Scale, three studies used the Self-Rating Depression Scale, three studies used the Geriatric Depression Scale, two studies used the Patient Health Questionnaire, two studies used the second version of the Beck Depression Inventory, and one study used the Symptom Checklist 90 to measure depression. A significant effect in favor of multi-component intervention over the control group was found [SMD = −0.29, 95% CI (−0.46, −0.11), *p* = 0.001, I^2^ = 0%]. The study not included in the meta-analysis also reported that multi-component intervention was statistically significant in reducing the depressive symptoms of caregivers [46].

Removing a trial [19] with the largest sample size did not change the result (*p* = 0.003). Furthermore, when we classified studies by the type of participants, multi-component interventions involving caregiver only as participants [SMD = −0.42, 95% CI (−0.62, −0.22), *p <* 0.001, I^2^ = 43%] were associated with slightly larger and statistically significant reductions in depressive symptoms than dyadic interventions [SMD = −0.09, 95% CI (−0.32, 0.14), *p* = 0.42, I^2^ = 68%]. Subgroup analysis according to intervention delivery methods revealed that the difference between groups was not significant (*p* = 0.46) (Table S4). Seven studies [20,24,36,40,41,42,47] were pooled to examine the effectiveness of the multi-component interventions on the depression of informal caregivers at three-month follow-up and no significant difference between groups was found (*p* = 0.26).

#### 3.5.2. Impact on the Caregiver’s Burden

Twelve studies were pooled for the post-intervention burden of informal caregivers, with a small to moderate effect being detected [SMD = −0.34, 95% CI (−0.53, −0.16), *p* = 0.0003, I^2^ = 60%] (Figure S2). Sensitivity analysis excluding one study with the largest sample size had no significant effect on the combined effect size [19]. No significant difference was detected between studies that worked with caregivers only or that included dyadic intervention (*p* = 0.05). However, the difference between face-to-face and online delivery methods was significant (*p* = 0.02) (Table S4). Due to the extremely high heterogeneity (I^2^ = 95%), meta-analysis at three-month follow-up was not conducted for the six studies providing sufficient data. Four of the six studies reported caregiver burden was significantly reduced at three-month follow-up [20,24,46,47].

#### 3.5.3. Impact on the Caregiver’s Anxiety

We pooled seven studies evaluating the effects of multi-component interventions on the anxiety of informal caregivers (Figure S2). Overall, the effect was statistically significantly different between the intervention and control groups [SMD = −0.53, 95% CI (−0.78, −0.27), *p* < 0.001, I^2^ = 48%]. Sensitivity analysis was performed by omitting one study in each round and the results did not change. However, no significant difference was detected between studies using face-to-face and online delivery methods (*p* = 0.35), although the only study which used online intervention, reported a much larger effect [21] (Table S4). Three highly heterogeneous (I^2^ = 98%) studies reported anxiety outcomes for the third month after the intervention, but no statistically significant difference was found.

#### 3.5.4. Impact on the Caregiver’s Stress

Five studies reported the effects of multi-component interventions on caregiver stress (Figure S2), and the results revealed that the overall effect was not significant [SMD = −0.23, 95% CI (−0.47, 0.01), *p* = 0.06, I^2^ = 0%]. Subgroup analysis showed no significant difference between studies with caregivers only or dyadic studies (*p* = 0.95) (Table S4). Excluding a study by Cristancho-Lacroix [42], multi-component interventions tended to reduce the stress of caregivers, although this was of borderline statistical significance [SMD = −0.28, 95% CI (−0.55, −0.01), *p* = 0.04, I^2^ = 0%]. Three studies [20,41,42] extended the follow-up time by three months, but no significant changes were observed (*p* = 0.61).

#### 3.5.5. Publication Bias

Publication bias were assessed using funnel plots (Figure S3 in the Supplement) and Eggers’ test, which did not indicate the presence of funnel plot asymmetry or publication bias in caregiver subjective well-being (*p* = 0.25), depression (*p* = 0.22), burden (*p* = 0.18), anxiety (*p* = 0.95) and stress (*p* = 0.57).

## 4. Discussion

Informal caregivers of people with dementia bear a heavy burden of care and increase their risk of physical and mental illnesses [48]. Caregiver support is one of the seven action areas in the Global Action Plan on the Public Health Response to Dementia 2017–2025 developed by the World Health Organization [49]. Multi-component interventions are consistently reported as the most effective intervention for maintaining caregiver health, providing caregivers with a variety of comprehensive support designed to meet their individual needs [50]. However, there is an inconclusive report on the impact of multi-component interventions on the well-being of caregivers. This systematic review synthesized all available evidence in the literature and identified 31 studies involving 3939 informal caregivers of people with dementia. Significant results were found for subjective well-being, depression symptoms, burden, and anxiety.

In contrast to a recent review [28], this review showed that multi-component interventions significantly improved the subjective well-being of the informal caregivers and the effect size was between small and moderate. One possible explanation is that the former study included randomized and nonrandomized studies, but this review only included higher-quality RCTs, and the number of studies included in this review increased, expanding the sample size. The difference may also be caused by the different emphasis on the intervention content included in the original study. The original research interventions included in the previous systematic review were psychosocial interventions and multicomponent interventions targeting the experience and/or behavior of informal caregivers. However, well-being does not only involve psychology, but also social, spiritual, and emotional factors [6]. The research included in this review can be a multi-component intervention with no limitations on the content of the intervention. The next step of research needs to develop standardized, unified, and robust subjective well-being measurement tools based on the concept map of well-being of informal caregivers proposed by existing studies [8]. Despite the promising quantitative finding of multi-component interventions on the positive aspect of well-being among caregivers, more independent replication and long-term follow-up are still needed.

Our results also showed that multi-component interventions could significantly reduce depression and anxiety symptoms of informal caregivers and alleviate care burden, which is consistent with those of previous reviews [28,51]. However, the effect was short-term, and the follow-up results were not statistically significant. Due to the small sample, the interpretation of the results should be cautious. In this study, 16 of the 17 studies that explored the impact of multi-component intervention on depression were personalized support based on the assessment results, needs or preferences of caregivers. Future research is needed, focusing on the impact of individually designed multi-component interventions on the depression and anxiety of caregivers. The care problems faced by informal caregivers of people with dementia are dynamic, varying from person to person, and cannot be generalized [48]. As caregivers become more experienced, their needs for the health of emerging issues and changing care recipients become more specific, and they are eager to obtain information about emerging issues [52]. Research has found that personally tailored activities can help increase participants’ positive emotions and have a positive impact on the burden and happiness of caregivers [53]. Therefore, it is vital to modify interventions for specific stages of the care trajectory faced by different caregivers [52,54]. 

Subgroup analysis found that for the subjective well-being, depression, burden, and anxiety of dementia caregivers, the effect of the face-to-face method might be better than the online method, which is inconsistent with previous studies [53]. This may be related to the fact that most of the participants included in this review were middle-aged and elderly adults who might not use computers [55]. By contrast, for the younger generation of caregivers, the independent time and place of the online intervention make it easier for them to get help [56]. Furthermore, the online intervention could expand the accessibility of the interventions, especially in the situation of COVID-19. Appropriate delivery methods should be considered according to the intervention content and target population to enhance the feasibility and effectiveness. With regard to the participants, findings of the subgroup analysis indicated that multi-component interventions recruiting caregivers only might have a better effect than dyadic interventions in terms of depression and burden. One possible reason is that intervention contents are mainly developed for caregivers [57], and caregivers can temporarily leave the caring environment and receive a short respite [25]. More detailed information about the intervention content and implementation strategies are required for exploring the best combination of intervention content and dosage in multi-component interventions.

Interventionists should have structured knowledge or professional experience in dementia [58]. Fifteen of the included 31 studies in this review were led or assisted by nurses, and nine of them were completed by multidisciplinary teams. It is reported that rapport and a higher sense of trust between the interventionist and the caregiver are key factors that facilitate the intervention, which helps to produce better results and a higher level of adherence [59]. Primary health professionals in the community are in an ideal position to provide multi-component interventions and coordinate services for informal caregivers due to the close contact, familiarity, and trust between them and caregivers [60,61]. However, some primary health professionals are not yet well prepared to undertake roles due to the lack of opportunities to engage in dementia care education [62]. It is recommended to rely on the existing public health services to deliver dementia training for health professionals and to integrate the available dementia care resources on the basis of communities or villages, which would help more healthcare professionals to provide tailored recommendations and support for people with dementia and informal caregivers [63].

The mechanisms by which multicomponent interventions improve caregiver well-being may be complex and multifactorial [64]. As previous research has illustrated, dementia care is a long-term process that forces informal caregivers to continually revise their coping strategies as the person with dementia goes through different stages of the disease, in which the caregiver’s needs are constantly changing and vary from person to person [65]. Caregiver well-being is a multiple concept that includes emotional, psychological, social, and spiritual aspects, and multi-component interventions contain a variety of components covering educational, physical, psychological, emotional, and social supports that can be selected by caregivers as needed and better meet caregiver’s needs, making it possible to personalize interventions and thus improve caregiver well-being [66]. In addition, multi-component interventions tend to have a longer intervention duration [67], and the ongoing contact between the intervention implementer and the caregiver provides continuity of care for the caregiver [68]. Caregivers reported access to ongoing care helped them seek out support services that were beneficial to them, and that “personal gains” were enhanced, such as inner strengths, self-confidence, and a sense of efficacy, which are important factors in promoting caregiver well-being [69].

This systematic evaluation extends previous research by providing evidence that supports the efficacy of multi-component interventions in improving positive and negative aspects of well-being for informal caregivers of people with dementia. As the aging of the global population accelerates, there is an urgent need for healthcare providers to identify effective ways to support informal dementia caregivers in community settings. Trained primary health professionals who integrate community resources and provide multifaceted support for caregivers can maximize the coverage of factors that meet the individual needs of most caregivers and help informal caregivers caring for people with dementia to effectively cope with the caregiving issues they face. This would help to address the concerns of the growing strain on the dementia-related health and social care systems in countries facing a rapidly aging population.

## 5. Limitations and Implications for Future Research

There are several limitations to this systematic review. The duration and content of the interventions in the included studies are diverse. What we extracted was the data measured immediately after the intervention and did not account for differences between groups at baseline. However, because the duration of the interventions ranged from 2 h to 48 weeks, the time point for the outcome measurement varied from study to study. This may have led to the different effect sizes reported by studies. Furthermore, most of the included studies failed to report follow-up data and a small number of trials were included in the meta-analysis to estimate the pooled follow-up effect, reducing the power of the analysis. Future studies exploring the best intervention dosage involving cost-effectiveness analysis and the most appropriate combination of different components in multi-component interventions with longer follow-up are therefore highly recommended. Due to the high heterogeneity and inadequate data, we were unable to estimate the overall effects of multi-component intervention on relationship satisfaction, empathy, and competence. Future research should incorporate more positive outcome measures.

## 6. Conclusions

This systematic review suggests that the integrated and diverse intervention components of multi-component interventions may help meet the changing needs of different caregivers during the progressive stages of the disease and effectively improve subjective well-being and reduce depression, anxiety, and burden among informal caregivers of people with dementia. Primary health professionals are important for dementia caregiving support, and they should be trained to better leverage their strengths of close contact and trust with caregivers to apply these effective multi-component interventions to informal caregivers of people with dementia. It is strongly suggested that intervention practice should focus on pre-intervention assessment and delivery methods should be tailored according to caregivers’ personal situations and preferences. Considering the lack of studies exploring the impact of multi-component interventions on caregiver well-being, especially the positive aspects, more rigorous RCTs incorporating more positive outcomes and with longer follow-up time are needed. It is also recommended that future studies have detailed information on the interventions to explore the best combination of the different components when practiced in the community.

## Data Availability

All data generated or analyzed during this study are included in this article and supplementary materials. Further inquiries can be directed to the corresponding author.

## Supplementary Materials

The following supporting information can be downloaded at: https://www.mdpi.com/article/10.3390/ijerph19126973/s1, Table S1: PRISMA 2020 Checklist; Table S2: The full electronic search strategy for the databases; Table S3: Characteristics of included studies, References [70,71,72,73,74,75,76,77,78,79,80] are cited in the supplementary materials; Table S4: The results of subgroup analysis by delivery methods and participant types; Figure S1: Risk of bias in included studies; Figure S2: Effect of multi-component interventions on informal caregiver’s burden, anxiety and stress (post-intervention); Figure S3: Funnel plots assessing publication bias on the meta-analysis of multicomponent intervention on informal caregiver outcomes (i.e., subjective well-being, depression, burden, anxiety, stress).
