# Supplementary material for: The Effectiveness of Multi-Component Interventions on the Positive and Negative Aspects of Well-Being among Informal Caregivers of People with Dementia: A Systematic Review and Meta-Analysis
Source: Int J Environ Res Public Health. 2022 Jun 7;19(12):6973. doi: 10.3390/ijerph19126973 (PMC9222573; doi:10.3390/ijerph19126973)
Supplement: Supplementary file 1 [file ijerph-19-06973-s001.zip › Figure S2_Effect of multi-component interventions on informal caregiverí»s burden, anxiety and stress (postintervention).pdf]

Burden

Study or Subgroup	Intervention Mean	Intervention SD	Intervention Total	Control Mean	Control SD	Control Total	Weight	Std. Mean Difference IV, Random, 95% CI
**2.2.1 faca-to-face**								
Berwig 2017	-0.43	8.41	41	7.05	8.09	40	8.0%	-0.90 [-1.36, -0.44]
Chien 2011	56.9	14.8	46	64	13.1	46	8.8%	-0.50 [-0.92, -0.09]
Gonyea 2006	16.55	7.39	40	18.77	7.94	40	8.3%	-0.29 [-0.73, 0.15]
Kor 2019	37.17	16.57	18	40.72	14.15	18	5.3%	-0.23 [-0.88, 0.43]
Yao 2011-Chinese	42.89	15.14	44	43.45	13.9	45	8.8%	-0.04 [-0.45, 0.38]
Yoo 2019	32.5	14.4	19	49.9	16.3	19	4.9%	-1.11 [-1.80, -0.42]
Zhu 2016-Chinese	42.27	10.6	62	48.83	10.13	60	9.8%	-0.63 [-0.99, -0.26]
**Subtotal (95% CI)**			**270**			**268**	**53.9%**	**-0.50 [-0.76, -0.25]**
Heterogeneity: Tau² = 0.06; Chi² = 12.68, df = 6 (P = 0.05); I² = 53%								
Test for overall effect: Z = 3.83 (P = 0.0001)								
**2.2.2 online**								
Belle 2006	17	8	96	17.7	9.6	86	11.3%	-0.08 [-0.37, 0.21]
Cristancho-Lacroix 2015	38.3	14.9	25	33.5	15.3	24	6.4%	0.31 [-0.25, 0.88]
Hattink 2015	2.43	0.98	27	2.8	0.96	32	7.1%	-0.38 [-0.89, 0.14]
Metcalfe 2019	11.8	6.69	30	14.9	7.3	31	7.2%	-0.44 [-0.95, 0.07]
Possin 2019	-1.9	11.86	426	0	11.86	229	14.1%	-0.16 [-0.32, 0.00]
**Subtotal (95% CI)**			**604**			**402**	**46.1%**	**-0.15 [-0.31, 0.01]**
Heterogeneity: Tau² = 0.01; Chi² = 4.79, df = 4 (P = 0.31); I² = 17%								
Test for overall effect: Z = 1.84 (P = 0.07)								
**Total (95% CI)**			**874**			**670**	**100.0%**	**-0.34 [-0.53, -0.16]**
Heterogeneity: Tau² = 0.06; Chi² = 27.19, df = 11 (P = 0.004); I² = 60%								
Test for overall effect: Z = 3.62 (P = 0.0003)								
Test for subgroup differences: Chi² = 5.26, df = 1 (P = 0.02), I² = 81.0%								

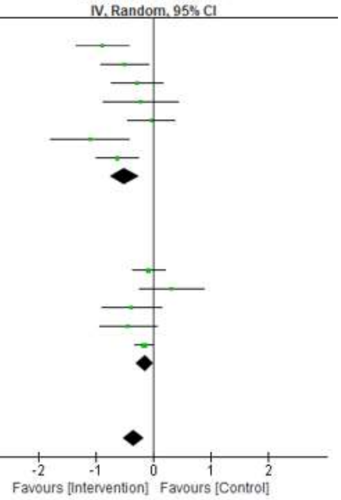

Anxiety

Study or Subgroup	Intervention Mean	Intervention SD	Intervention Total	Control Mean	Control SD	Control Total	Weight	Std. Mean Difference IV, Random, 95% CI
**2.3.1 face-to-face**								
Berwig 2017	-0.34	2	41	-0.03	1.98	40	16.4%	-0.15 [-0.59, 0.28]
Fei 2009-Chinese	1.44	0.43	31	1.77	0.49	34	14.2%	-0.71 [-1.21, -0.20]
Huang 2015-Chinese	25.36	4.23	10	27.14	3.28	10	6.5%	-0.45 [-1.34, 0.44]
Kor 2019	7.5	4.46	18	8.06	2.58	18	10.2%	-0.15 [-0.80, 0.50]
Liu 2007-Chinese	29.67	6.18	18	35.59	6.39	22	10.1%	-0.92 [-1.58, -0.26]
Luo 2012-Chinese	30.11	8.23	76	37.33	7.95	76	20.6%	-0.89 [-1.22, -0.55]
**Subtotal (95% CI)**			**194**			**200**	**77.9%**	**-0.57 [-0.87, -0.26]**
Heterogeneity: Tau² = 0.07; Chi² = 9.87, df = 5 (P = 0.08); I² = 49%								
Test for overall effect: Z = 3.63 (P = 0.0003)								
**2.3.2 online**								
Blom 2015	-1.69	3.26	90	-0.47	3.41	85	22.1%	-0.36 [-0.66, -0.07]
**Subtotal (95% CI)**			**90**			**85**	**22.1%**	**-0.36 [-0.66, -0.07]**
Heterogeneity: Not applicable								
Test for overall effect: Z = 2.39 (P = 0.02)								
**Total (95% CI)**			**284**			**285**	**100.0%**	**-0.53 [-0.78, -0.27]**
Heterogeneity: Tau² = 0.05; Chi² = 11.60, df = 6 (P = 0.07); I² = 48%								
Test for overall effect: Z = 4.07 (P < 0.0001)								
Test for subgroup differences: Chi² = 0.87, df = 1 (P = 0.35), I² = 0%								

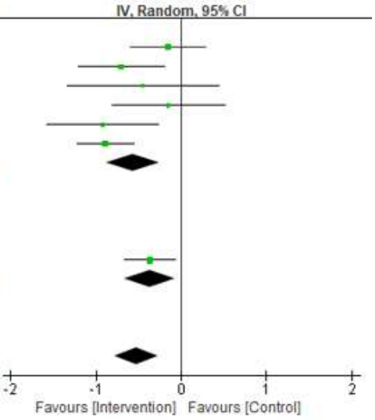

Stress

Study or Subgroup	Intervention Mean	Intervention SD	Intervention Total	Control Mean	Control SD	Control Total	Weight	Std. Mean Difference IV, Fixed, 95% CI
**2.5.1 face-to-face**								
Kor 2019	24.1	8.12	18	25.89	5.23	18	13.5%	-0.26 [-0.91, 0.40]
Pan 2020-Chinese	39.65	12.35	31	44.35	13.67	32	23.4%	-0.36 [-0.85, 0.14]
Prick 2015	5.67	2.36	27	5.85	2.13	32	22.1%	-0.08 [-0.59, 0.43]
**Subtotal (95% CI)**			**76**			**82**	**59.0%**	**-0.23 [-0.54, 0.08]**
Heterogeneity: Chi² = 0.58, df = 2 (P = 0.75); I² = 0%								
Test for overall effect: Z = 1.43 (P = 0.15)								
**2.5.2 online**								
Cristancho-Lacroix 2015	23.7	9.2	25	23.8	6.2	24	18.5%	-0.01 [-0.57, 0.55]
Metcalfe 2019	15.6	8.03	30	18.6	6.3	31	22.5%	-0.41 [-0.92, 0.10]
**Subtotal (95% CI)**			**55**			**55**	**41.0%**	**-0.23 [-0.61, 0.14]**
Heterogeneity: Chi² = 1.07, df = 1 (P = 0.30); I² = 6%								
Test for overall effect: Z = 1.21 (P = 0.23)								
**Total (95% CI)**			**131**			**137**	**100.0%**	**-0.23 [-0.47, 0.01]**
Heterogeneity: Chi² = 1.65, df = 4 (P = 0.80); I² = 0%								
Test for overall effect: Z = 1.87 (P = 0.06)								
Test for subgroup differences: Chi² = 0.00, df = 1 (P = 0.99), I² = 0%								

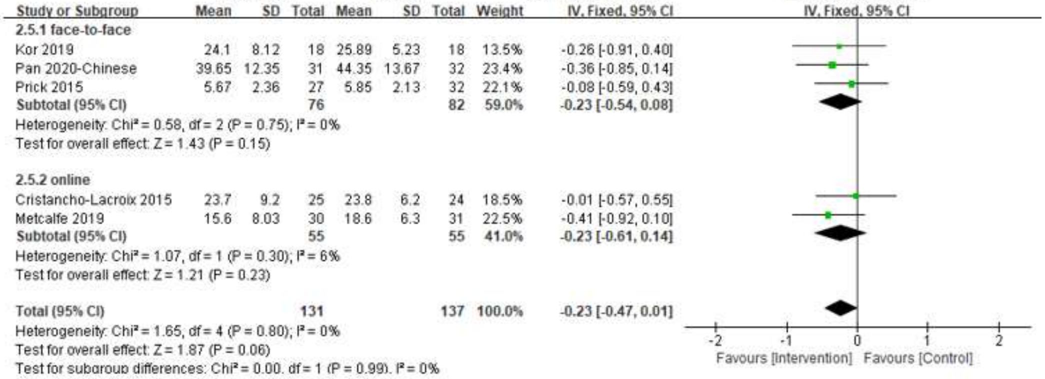

Note: CI = Confidence interval.

Figure S2. Effect of multi-component interventions on informal caregiver's burden, anxiety, and stress (postintervention).
