# Supplementary material for: The Effectiveness of Multi-Component Interventions on the Positive and Negative Aspects of Well-Being among Informal Caregivers of People with Dementia: A Systematic Review and Meta-Analysis
Source: Int J Environ Res Public Health. 2022 Jun 7;19(12):6973. doi: 10.3390/ijerph19126973 (PMC9222573; doi:10.3390/ijerph19126973)
Supplement: Supplementary file 1 [file ijerph-19-06973-s001.zip › Figure S3_Funnel plots.pdf]

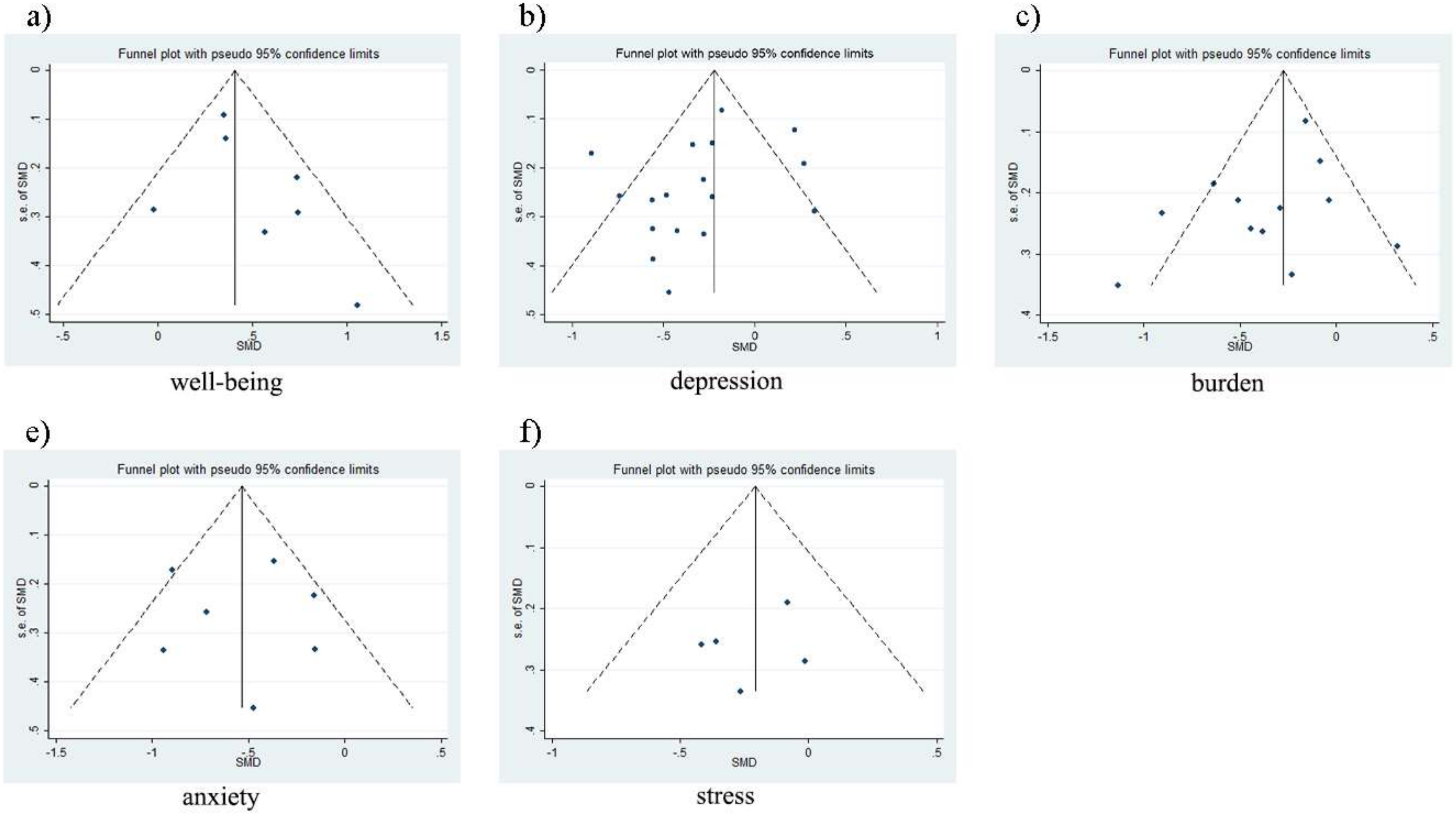

Figure S3. Funnel plots assessing publication bias on the meta-analysis of multicomponent intervention on informal caregiver outcomes (i.e. subjective well-being, depression, burden, anxiety, stress).
