# Supplementary material for: The Effectiveness of Multi-Component Interventions on the Positive and Negative Aspects of Well-Being among Informal Caregivers of People with Dementia: A Systematic Review and Meta-Analysis
Source: Int J Environ Res Public Health. 2022 Jun 7;19(12):6973. doi: 10.3390/ijerph19126973 (PMC9222573; doi:10.3390/ijerph19126973)
Supplement: Supplementary file 1 [file ijerph-19-06973-s001.zip › Table S2_The full electronic search strategy for the databases.pdf]

Table S2. The full electronic search strategy for the databases.

Search strategy-English databases

**Databases**	**Search strategy-date limits: prior to June 30, 2020**	**Items (-20200630)**
1.ALOIS	"multicomponent" OR "multi" OR "multidimensional" OR "combin intervention" OR "multimodel" OR "multifaceted" OR "multiple" OR "multifactorial" OR "multi-domain" OR "comprehensive"<br>Study Type： Intervention Study<br>Intervention type ： Any<br>Current Status： Completed<br>Study Design： RCT	4

Databases	Search strategy-date limits: prior to June 30, 2020	Items (-20200630)
	Study Aim: Caregiver Focused	
2.MEDLINE(OVID)	1. exp Dementia/	894
	2. Wernicke Encephalopathy/	
	3. Dementia, Amnestic/	
	4. dement*.mp.	
	5. alzheimer*.mp.	
	6. (lewy* adj2 bod*).mp.	
	7. (chronic adj2 cerebrovascular).mp.	
	8. ("organic brain disease" or "organic brain syndrome").mp.	

Databases	Search strategy-date limits: prior to June 30, 2020	Items (-20200630)
	9. (cerebr* adj2 deteriorat*).mp.	
	10. (cerebral* adj2 insufficient*).mp.	
	11. (pick* adj2 disease).mp.	
	12. (creutzfeldt or jcd or cjd).mp.	
	13. binswanger*.ab.	
	14. huntington*.mp.	
	15. or/1-14	
	16. caregiver*.mp.	
	17. exp Caregiver/	

Databases	Search strategy-date limits: prior to June 30, 2020	Items (-20200630)
	18. 'informal caregiver*'.ti,ab.	
	19. carer*.mp.	
	20. spous*.ti,ab.	
	21. partner*.ti,ab.	
	22. family*.ti,ab.	
	23. relative*.ti,ab.	
	24. neighbor*.ti,ab.	
	25. or/16-24	
	26. multi?component*.ti,ab.	

Databases	Search strategy-date limits: prior to June 30, 2020	Items (-20200630)
	27. (multi* adj2 intervention*).ti,ab.	
	28. multi?domain.ti,ab.	
	29. multidimensional*.ti,ab.	
	30. comprehensive.ti,ab.	
	31. (combin* adj2 intervention*).ti,ab.	
	32. (multi* adj2 model).ti,ab.	
	33. multifaceted*.ti,ab.	
	34. multiple.ti,ab.	

Databases	Search strategy-date limits: prior to June 30, 2020	Items (-20200630)
	35. (pharmacological or pharmacology or "drug therapy" or drugs or psychopharmacology).mp.	
	36. or/26-34	
	37. 36 not 35	
	38. randomized controlled trial.pt.	
	39. controlled clinical trial.pt.	
	40. randomized.ab.	
	41. placebo.ab.	
	42. randomly.ab.	

Databases	Search strategy-date limits: prior to June 30, 2020	Items (-20200630)
	43. trial.ab.	
	44. groups.ab.	
	45. ("double-blind*" or "single-blind*").ti,ab.	
	46. "double?blind*".ti,ab.	
	47. (animals not (humans and animals)).sh.	
	48. or/38-46	
	49. 48 not 47	
	50. 15 and 25 and 37 and 49	
	51. English language and yr="1860 - 2020"	

Databases	Search strategy-date limits: prior to June 30, 2020	Items (-20200630)
3.EMBASE	1. 'dementia'/exp	1399
	2. 'Wernicke Encephalopathy*':ab,ti	
	3. amnestic:ab,ti	
	4. dement*:ab,ti	
	5. alzheimer*:ab,ti	
	6. 'lewy* bod*':ab,ti	
	7. 'chronic* cerebrovascular'	
	8. 'organic brain disease':ab,ti OR 'organic brain syndrome':ab,ti	
	9. 'cerebr* deteriorat*':ab,ti	

Databases	Search strategy-date limits: prior to June 30, 2020	Items (-20200630)
	10. ' cerebral* insufficient*':ab,ti	
	11. 'pick presenile dementia':ab,ti	
	12. binswanger*:ab,ti	
	13. huntington*:ab,ti	
	14. #1 OR #2 OR #3 OR #4 OR #5 OR #6 OR #7 OR #8 OR #9 OR #10 OR #11 OR #12 OR #13	
	15. caregiver/de	
	16. caregiver*:ab,ti	
	17. 'informal caregiver*':ab,ti	

Databases	Search strategy-date limits: prior to June 30, 2020	Items (-20200630)
	18. carer*:ab,ti	
	19. spous*:ab,ti	
	20. partner*:ab,ti	
	21. family*:ab,ti	
	22. relative*:ab,ti	
	23. neighbor*:ab,ti	
	24. #15 OR #16 OR #17 OR #18 OR #19 OR #20 OR #21 OR #22 OR #23	
	25. 'multi*component':ab,ti	
	26. 'multi* intervention*':ab,ti	

Databases	Search strategy-date limits: prior to June 30, 2020	Items (-20200630)
	27. 'multi*domain':ab,ti	
	28. multidimensional*:ab,ti	
	29. comprehensive:ab,ti	
	30. 'combin* intervention*':ab,ti	
	31. 'multi* model':ab,ti	
	32. multifaceted*:ab,ti	
	33. multiple:ab,ti	
	34. pharmacological OR pharmacology OR 'drug therapy' OR drugs OR psychopharmacology	

Databases	Search strategy-date limits: prior to June 30, 2020	Items (-20200630)
	35. #25 OR #26 OR #27 OR #28 OR #29 OR #30 OR #31 OR #32 OR #33	
	36. 35 NOT 34	
	37. 'randomized controlled trial':ab,ti	
	38. 'controlled clinical trial':ab,ti	
	39. randomized: :ab,ti	
	40. placebo:ab	
	41. randomly:ab	
	42. trial:ab	
	43. groups:ab	

Databases	Search strategy-date limits: prior to June 30, 2020	Items (-20200630)
	44. 'double* blind':ab	
	45. 'single* blind':ab	
	46. 'double blind*':ab,ti	
	47. #37 OR #38 OR #39 OR #40 OR #41 OR #42 OR #43 OR #44 OR #45 OR #46	
	48. 'humans and animals'	
	49. animal	
	50. 47 not 48	
	51. 50 not 49	

Databases	Search strategy-date limits: prior to June 30, 2020	Items (-20200630)
	52. #14 AND #24 AND #36 AND #51 AND ([chinese]/lim OR [english]/lim) AND [<1966-2020]/py	
4.LILACS	1. (dementia OR alzheimer$ OR demenc$ OR AD OR demência) AND (carer$ OR caregiver$ OR informal caregiver$ OR spous$ OR partner$ OR family OR relative$ OR neighbor$ OR MH:"caregiver" OR MH:"family") AND (multi$component$ OR multi$intervention$ OR multi$domain OR multidimensional OR comprehensive OR combin$intervention$ OR multi$model OR multifaceted OR multi$faceted OR multiple OR multi$factorial) [Words]	11

Databases	Search strategy-date limits: prior to June 30, 2020	Items (-20200630)
	2. randomly OR randomised OR randomized OR trial OR ensaio clínico [Words]	
	3. English [Language]	
	4. #1 AND #2 AND #3	
5.PsycINFO（OVID）	1. exp Dementia/	537
	2. exp Wernickes Syndrome/	
	3. dement*.mp.	
	4. alzheimer*.mp.	
	5. (lewy* adj2 bod*).mp.	
	6. (chronic adj2 cerebrovascular).mp.	

Databases	Search strategy-date limits: prior to June 30, 2020	Items (-20200630)
	7. ("organic brain disease" or "organic brain syndrome").mp.	
	8. (cerebr* adj2 deteriorat*).mp.	
	9. (cerebral* adj2 insufficient*).mp.	
	10. (pick* adj2 disease).mp.	
	11. (creutzfeldt or jcd or cjd).mp.	
	12. binswanger*.mp.	
	13. huntington*.mp.	
	14. or/1-13	
	15. caregiver*.mp.	

Databases	Search strategy-date limits: prior to June 30, 2020	Items (-20200630)
	16. exp caregivers/	
	17. (informal adj2 caregiver*).ti,ab.	
	18. carer*.mp.	
	19. spous*.ti,ab.	
	20. partner*.ti,ab.	
	21. family*.ti,ab.	
	22. relative*.ti,ab.	
	23. neighbor*.ti,ab.	
	24. or/15-23	

Databases	Search strategy-date limits: prior to June 30, 2020	Items (-20200630)
	25. multi*component*.mp.	
	26. (multi* adj2 intervention*).ti,ab.	
	27. multi*domain.mp.	
	28. multidimensional*.ti,ab.	
	29. comprehensive.ti,ab.	
	30. (combin* adj2 intervention*).ti,ab.	
	31. (multi* adj2 model).ti,ab.	
	32. multifaceted*.ti,ab.	
	33. multiple.ti,ab.	

Databases	Search strategy-date limits: prior to June 30, 2020	Items (-20200630)
	34. (pharmacological or pharmacology or "drug therapy" or drugs or psychopharmacology).mp.	
	35. or/25-33	
	36. 35 not 34	
	37. "randomized controlled trial".pt.	
	38. "controlled clinical trial".pt.	
	39. randomized.ab.	
	40. placebo.ab.	
	41. randomly.ab.	

Databases	Search strategy-date limits: prior to June 30, 2020	Items (-20200630)
	42. trial.ab.	
	43. groups.ab.	
	44. ("double-blind*" or "single-blind*").ti,ab.	
	45. "double blind*".ti,ab.	
	46. (animals not (humans and animals)).sh.	
	47. or/37-45	
	48. 47 not 46	
	49. 14 AND 24 AND 36 AND 48	
	50. English [Language]	

Databases	Search strategy-date limits: prior to June 30, 2020	Items (-20200630)
6.CINAHL（	S1. MH "dementia"	206
EBSCOhost）	S2. MH "Dementia, Amnestic"	
	S3. MH "Wernicke's Encephalopathy"	
	S4. AB dement*	
	S5. AB alzheimer*	
	S6. AB "lewy* N2 bod*"	
	S7. AB "chronic N2 cerebrovascular"	
	S8. AB "organic brain disease" or "organic brain syndrome"	
	S9. AB "cerebral* N2 insufficient*"	

Databases	Search strategy-date limits: prior to June 30, 2020	Items (-20200630)
	S10. AB "pick* N2 disease"	
	S11. AB binswanger*	
	S12. AB huntington*	
	S13. S1 OR S2 OR S3 OR S4 OR S5 OR S6 OR S7 OR S8 OR S9 OR S10 OR S11 OR S12	
	S14. MH caregiver	
	S15. AB caregiver*	
	S16. AB "informal caregiver*"	
	S17. AB spous*	

Databases	Search strategy-date limits: prior to June 30, 2020		Items (-20200630)
	S18.	AB partner*	
	S19.	AB famil*	
	S20.	AB relative*	
	S21.	AB neighbor*	
	S22.	S14 OR S15 OR S16 OR S17 OR S18 OR S19 OR S20 OR S21	
	S23.	TX "multi*component*"	
	S24.	TX "multi* N2 intervention*"	
	S25.	TX "multi*domain"	
	S26.	TX multidimensional*	

Databases	Search strategy-date limits: prior to June 30, 2020	Items (-20200630)
	S27. AB comprehensive	
	S28. AB "combin* intervention*"	
	S29. AB "multi* N2 model"	
	S30. AB multifaceted*	
	S31. AB multiple	
	S32. TX pharmacological OR pharmacology OR "drug therapy" OR drugs OR psychopharmacology	
	S33. S23 OR S24 OR S25 OR S26 OR S27 OR S28 OR S29 OR S30 OR S31	
	S34. S34 NOT S32	

Databases	Search strategy-date limits: prior to June 30, 2020		Items (-20200630)
	S35.	TX "randomized controlled trial"	
	S36.	TX RCT	
	S37.	TX "controlled clinical trial"	
	S38.	AB placebo*	
	S39.	AB randomized	
	S40.	AB randomised	
	S41.	AB randomly	
	S42.	AB groups OR "control group"	
	S43.	AB "double-blind*" OR "single-blind*"	

Databases	Search strategy-date limits: prior to June 30, 2020	Items (-20200630)
	S44. AB "double#blind*"	
	S45. S35 OR S36 OR S37 OR S38 OR S39 S40 OR S41 OR S42 OR S43 OR S44	
	S46. S13 AND S22 AND S34 AND S45	
	S47. Published Date: -20200630;	
	S48. English Language	
7.Web of science	#1. TS=(Alzheimer OR dementia* OR Alzheimer* OR "AD" OR "Wernicke Encephalopathy*" OR amnestic OR Binswanger OR huntington OR lewy OR "pick presenile" OR "cerebr* deterioration" OR "organic brain disease" OR "organic brain syndrome")	1196

Databases	Search strategy-date limits: prior to June 30, 2020	Items (-20200630)
	#2. TS=(carer* OR caregiver* OR "informal caregiver*" OR spouse OR partner OR famil* OR relative* OR neighbor*)	
	#3. TS=("multi*component*" OR "multi* intervention*" OR "multi*domain" OR multidimensional OR comprehensive OR "combin* intervention*" OR "multi*model" OR multifaceted OR "multi*faceted" OR multiple OR "multi*factorial")	
	#4. TS=(pharmacological OR pharmacology OR "drug therapy" OR drugs OR psychopharmacology)	
	#5. #3 not #4	

Databases	Search strategy-date limits: prior to June 30, 2020	Items (-20200630)
	#6. TS=("randomized controlled trial" OR randomized OR randomised OR randomly OR trial OR RCT OR placebo OR "control group" OR "double-blind*" "double blind*"OR "single-blind*")	
	#7. TS=(animals not (humans and animals))	
	#8. #6 not #7	
	#9. #1 AND #2 AND #5 AND #8	
	#10.Timespan=-20200630	
	#11.Search language=English	

Search strategy-Chinese databases

Databases	Search strategy-date limits: prior to June 30, 2020	Items(-20200630)
Wanfang (万方)	题名或关键词:(痴呆 OR 阿尔茨海默 OR 阿尔兹海默 OR 失智症 OR 认知症)*题名或关键词:(照顾者 OR 照护者 OR 配偶 OR 家属 OR 家人 OR 儿女 OR 子女 OR 亲属 OR 朋友 OR 邻居)*题名或关键词:(干预 OR 治疗 OR 训练 OR 培训 OR 疗法 OR 支持 OR 多模式照护)	238
CBM (中国生物医学文献服务系统)	(("痴呆"[核心字段:智能] OR "阿尔茨海默"[核心字段:智能] OR "阿尔兹海默"[核心字段:智能] OR "失智症"[核心字段:智能] OR "认知症"[核心字段:智能]) AND("照顾者"[核心字段:智能] OR "照护者"[核心字段:智能] OR "配偶"[核心字段:智能] OR "家属"[核心字段:智能] OR "家人"[核心字段:智能] OR "儿女"[核心字段:智能] OR "子女"[核心字段:智能]OR "亲属"[核心字段:智能] OR "朋友"[核心字段:智能] OR "邻居"[核心字段:智能]) AND ("干预"[核心字段:智能] OR "治疗"[核心字段:智能] OR "	182

Databases	Search strategy-date limits: prior to June 30, 2020	Items(-20200630)
	训练"[核心字段:智能] OR "培训"[核心字段:智能] OR "疗法"[核心字段:智能] OR "支持"[核心字段:智能] OR "多模式照护"[核心字段:智能])) AND -2020[日期]	
VIP (维普)	((((题名或关键词=痴呆 OR 题名或关键词=阿尔茨海默) OR 题名或关键词=阿尔兹海默) OR 题名或关键词=失智症) OR 题名或关键词=认知症) AND (((((((题名或关键词=照顾者 OR 题名或关键词=照护者) OR 题名或关键词=配偶) OR 题名或关键词=家属) OR 题名或关键词=家人) OR 题名或关键词=儿女) OR 题名或关键词=子女)OR 题名或关键词=亲属) OR 题名或关键词=朋友) OR 题名或关键词=邻居)) AND (((题名或关键词=干预 OR 题名或关键词=治疗) OR 题名或关键词=训练) OR 题名或关键词=培训 OR 题名或关键词=疗法) OR 题名或关键词=支持) OR 题名或关键词=多模式照护))	141

Databases	Search strategy-date limits: prior to June 30, 2020	Items(-20200630)
CNKI (中国知网)	(TI=('痴呆' + '阿尔茨海默' + '阿尔兹海默' + '失智症' + '认知症') OR KY=('痴呆' + '阿尔茨海默' + '阿尔兹海默' + '失智症' + '认知症')) AND (TI=('照顾者' + '照护者' + '配偶' + '家属' + '家人' + '儿女' + '子女' + '亲属' + '朋友' + '邻居') OR KY=('照顾者' + '照护者' + '配偶' + '家属' + '儿女' + '亲属' + '朋友' + '邻居')) AND (TI=('干预' + '治疗' + '训练' + '培训') OR KY=('干预' + '治疗' + '训练' + '培训' + '疗法' + '支持' + '多模式照护'))<br>中文文献<br>发表时间：-2020-06-30	138
