# Supplementary material for: The Effectiveness of Multi-Component Interventions on the Positive and Negative Aspects of Well-Being among Informal Caregivers of People with Dementia: A Systematic Review and Meta-Analysis
Source: Int J Environ Res Public Health. 2022 Jun 7;19(12):6973. doi: 10.3390/ijerph19126973 (PMC9222573; doi:10.3390/ijerph19126973)
Supplement: Supplementary file 1 [file ijerph-19-06973-s001.zip › Table S3. Characteristics of included studies.pdf]

Table S3. Characteristics of included studies.

Author (Year)	Country	Participants	Intervention	Control	Outcomes	Follow-up
(Au, et al., 2014)[1]	China (Hong Kong)	Informal family caregivers<br>Int: 58.1, 78.6% female<br>Con:55.1, 77.4% female	Telephone-assisted pleasant-event scheduling (TAPES)：behavioral activation and the pleasant event schedule + six subsequent telephone calls of 20 minutes each +adaptive coping and regular weekly meetings<br>1month (N=30)	Usual care (N=30)	1.Depression: CES-D<br>2.Self-Efficacy: RSCSE	Postintervention
(Au, et al., 2015) [2]	China (Hong Kong)	Informal family caregivers<br>Int: 55.2, 93.1% female<br>Con: 57.9, 73.3% female	PsyED-BA<br>Four weeks psychoeducation program telephone calls lasting 30 minutes (information +stress coping + pleasant events + communication) +behavioral activation (Eight biweekly	PsyED: the materials of the psychoeducation* program<br>(N=30)	1.Depression: CES-D (22)<br>2.Relationship satisfaction: RAS	Postintervention

			telephone call lasting 15 to 20 minutes) +social work services<br>5months(N=29)			
(Belle, et al., 2006) [3]	United States	Informal family caregivers<br>Int: 63.2, 81.4% female<br>Con: 63.5, 81.2% female<br>+care recipients: Alzheimer disease or relative disorders<br>Int: 78.6, 54.7% female<br>Con: 77.5, 45.8% female	REACH-(Dyadic):<br>Information +didactic instruction +role playing +problem solving +skills training +stress management techniques +telephone support groups<br>6 months(N=96)	Education materials +2 brief telephone "check-in" calls (N=86)	1.Depression: CES-D (10)<br>2.Burden: ZBI (12)	Postintervention
(Berwig, et al., 2017) [4]	German	Informal caregivers<br>Int: 72.3, 68.0% female<br>Con: 74.0, 64.0% female	DE-REACH intervention:<br>Information transfer +psycho-education +role-playing +problem-solving +practice of skills +techniques for stress management +caregiver notebook +local support group	Usual care (N=40)	1.Burden: ZBI (22)<br>2.Anxiety: PHQ-4<br>3.Depression: PHQ-4	Postintervention<br>9 months

			6 months (N=41)			
(Birkenhäger-Gillesse, et al., 2020) [5]	Netherlands	Family caregivers:<br>Int: 72.5, 76.3% female<br>Con: 73.2, 74.0% female<br>People with dementia:<br>Int: 76.3<br>Con: 77.6	(Dyadic)<br>14 Psycho-educational sessions (isolation + medical aspects of dementia + planning for the future + re-rolling + reminiscence and orientation + communication + provide strategies to cope with criticism + activities +safety + nursing skills+ exercise + nutrition + self-care + using community services)<br>5 days (N=49)	Usual care (N=38)	1.Satisfaction of life: VAS<br>2.Burden: Self-Rated Burden Scale-VAS<br>3.Depression: CES-D<br>4.Anxiety: HADS-A	3 months
(Blom, et al., 2015) [6]	Netherlands	Family caregivers:(CES-D>4 or HADS-A>3 or a burden score>6 on a scale ranging from 0 to 10)<br>Int: 61.5, 69.8% female<br>Con: 60.8, 68.8% female	Internet course Mastery over Dementia (MoD): problem solving +cognitive restructuring +assertiveness training +homework<br>6 months (N=90)	A minimal intervention (e-bulletins)<br>(N=85)	1.Depression: CES-D (20)<br>2.Anxiety: HADS-A (7)	Postintervention

(Chien, et al., 2011) [7]	China (HongKong)	Family caregivers<br>Int: 44.9, 67.4% female<br>Con: 45.6, 65.2% female	1 month need's assessment +5 months sessions (education, sharing and discussion, psychological<br>support and problem-solving)<br>6 months (N=46)	Usual care<br>(N=46)	1.Burden: FCBI	Postintervention<br>12 months<br>18 months
(Cristancho-Lacroix, et al., 2015) [8]	France	Informal caregivers (scored 12 or more on the Perceived Stress Scale)<br>Int: 64.2, 64.0% female<br>Con: 59.0, 67.0% female	Web-based program (once a Week)<br>(education +skill training +coping with behavioral +social and financial support +peer support)<br>3 months (N=25)	Usual care<br>(N=24)	1.Stress: PSS-14<br>2.Self-efficacy: RSCSE<br>3.Subject burden: ZBI<br>4.Depression: BDI-II<br>5.Self-perceived emotion: NHP<br>6. Relationship satisfaction: VAS	Postintervention<br>6 months

(Duggleby, et al., 2018) [9]	Canada	Informal caregivers<br><br>Int: 63.4, 79.0% female<br><br>Con: 63.9, 84.0% female	Web-based intervention-MT4C:<br><br>(1) about me (2) common changes to expect (3) frequently asked questions (4) resources (5) important health information (6) calendar<br><br>3 months (N=78)	Usual care (N=89)	1.Self-efficy: GSES	Postintervention
(Elliott, et al., 2010) [10]	United States	Family caregivers and close friends<br><br>Int: 62.3, 81.8% female<br><br>Con: 60.2, 85.8% female	REACH (Dyadic):<br><br>Information +didactic instruction +role playing +problem solving +skills training +stress management techniques +telephone support groups<br><br>6 months (N=257)	Education materials +2 brief telephone "check-in" calls (N=238)	1.Mood improvement: questionnaire-mood improvement	Postintervention
(Fei, et al., 2009) [11]	China	Relative caregivers<br><br>Int: 54.5, 77.4% female<br><br>Con: 56.1, 64.7% female	Assessment +knowledge and skill guidance +pressure identification and regulation<br><br>3 months (N=31)	Usual care (N=34)	1.Depression (SCL-90)<br><br>2.Anxiety (SCL-90)	Postintervention

(Gitlin, et al., 2010) [12]	United States	Family caregivers<br>Int: 62.4, 90.7% female<br>Con: 62.0, 87.3% female	The COPE program (Dyadic):<br>Assessments +caregiver education (patient capabilities, potential effects of medications, pain, constipation, dehydration) +reduce stress<br>4 months (N=102)	Usual care (N=107)	1.Well-being: The 13-item Perceived Change Index	Postintervention
(Gonyea, et al., 2006) [13]	United States	Family caregivers<br>Mean age: 64.6, 67% female	Behavioral intervention (behavior therapy and behavioral activation) +discuss personal challenges +peer support +respite care<br>5 weeks (N=40)	Psychoeducational intervention*<br>(N=40)	1.Burden: ZBI (12)	Postintervention
(Han, et al., 2020) [14]	Korea	Relative caregivers<br>Int: 62.8, 83.3% female<br>Con: 59.4, 85.2% female	Dementian Live™ (The simulation-based empathy enhancement program) +The lecture-based education (information +communication strategies + stress management strategies)	The lecture-based education (N=27)	1.Well-being: (CarerQol-VAS)	Postintervention

			only once (N=24)			
(Hattink, et al., 2015) [15]	Netherlands and the United Kingdom	Informal caregivers<br>Int: 52.9, 74.0% female<br>Con: 55.0, 69.0% female	STAR E-Learning course:<br>Dementia information +problem solving strategies +adaptation and coping influences behavior and mood +support strategies + positive and empathic communication<br>4 months (N=27)	Wait-list group<br>(N=32)	1.Empathy: IRI<br>2.Burden: questionnaire<br>3.Competence: SSCQ	Postintervention
(Hepburn, et al., 2001) [16]	United States	Relative caregivers<br>Mean age: 65.0, 70.0% female	(Group Settings)<br>Information provision +concept development +role clarification +belief clarification +mastery-focused coaching<br>2 months (N=60)	Wait-list group (N=34)	1.Depression: CES-D (20)<br>2.Burden: ZBI (22)	5 months
(Huang, 2015) [17]	China	Informal caregivers	Cognitive behavior intervention +health education +social support	Usual care (N=10)	1.Depression (SDS)<br>2.Anxiety (SAS)	Postintervention (In the ninth month)

			12 months (N=10)		3.Positive aspects of caregiving	
(Kor, et al., 2019) [18]	China (Hong Kong)	Family caregivers<br>Int: 58.2, 77.8% female<br>Con: 56.0, 88.9%	(Group setting)<br>Mindfulness activities +psychoeducation +group sharing<br>10 weeks (N=18)	Usual care (N=18)	1.Stress: PSS<br>2.Burden: ZBI<br>3.Depression: CES-D<br>4.Anxiety: HADS-A<br>5.Resilience: BRS	Postintervention<br>3 months
(Laakkonen, et al., 2016) [19]	Finland	Spouse caregivers<br>Int: 75.9, 64.2% female<br>Con: 73.8, 60.9% female	(Dyadic)<br>Enhance self-efficacy and problem-solving skills and to provide peer<br>support.<br>2 months (N=67)	Usual care (N=67)	1.Competence: SCQ	Postintervention (In the third month)<br>9 months
(Liu, et al., 2007) [20]	China	Family caregivers<br>Int: 72.8	Knowledge and skills guidance +cognitive function exercise	Usual care (N=22)	1.Depression: SDS<br>2.Anxiety: SAS	Postintervention

		Con: 71.7	+pressure identification and regulation<br>12 months (N=18)			
(Luo, et al., 2012) [21]	China	Family caregivers	Knowledge education +care Skills Instruction + psychology guidance<br>12 months (N=76)	Usual care (N=76)	1.Depression: SDS<br>2.Anxiety: SAS	Postintervention
(Marriott, et al., 2000) [22]	United Kingdom	Family caregivers (with a score on the General Health Questionnaire of 5 or above)<br>Int: 69.6, 64.2% female<br>Con: 58.1, 78.6% female	(Dyadic)<br>Family intervention +four booklets<br>(education +stress management +coping skill training)<br>9 months (N=14)	Usual care (N=14)	1.Depression: BDI	Postintervention<br>12 months
(Metcalfe, et al., 2019) [23]	Six European countries<br>(England /France/	Informal caregivers<br>Int: 57.6, 60.0% female<br>Con: 57.2, 61.3% female	Online program:<br>(medical explanations + management of cognitive and behavioral symptoms +adapting to relationship changes	Wait-list control (N=31)	1.Self-efficacy: RSCSE<br>2.Stress: PSS<br>3.Burden: BSFC	Postintervention

	Germany/Netherlands/Portugal/Sweden)		+available care +support +self-care suggestions)<br>6 weeks (N=30)			
(Pan, et al., 2020) [24]	China	Family caregivers<br>Mean age: 54.5	(Dyadic)<br>Consulting + problems coping strategies + health education or related information about dementia<br>6 months (N=31)	Usual care (N=32)	1.Depression: GDS<br>2.Stress: RSS	Postintervention
(Possin, et al., 2019) [25]	United States	Informal caregivers<br>Int: 65.3, 69.9% female<br>Con: 64.0, 73.1% female	(Dyadic)<br>the Care Ecosystem: (Immediate needs +medication reconciliation and review +safety screen and recommendations +referrals and caregiver education +caregiver well-being +behavior management +advance care planning) monthly	Usual care (N=229)	1.Depression: PHQ-9<br>2.Burden: ZBI (12)<br>3.Self-efficacy: questionnaire	Postintervention

			12 months (N=426)			
(Prick, et al., 2015) [26]	Netherland	Family caregivers<br>Int: 73.0, 66.7% female<br>Con: 71.0, 77.8% female	(Dyadic)<br>Physical exercise + support (psychoeducation +communication skills training + pleasant activities training)<br>3 months (N=57)	Minimal intervention (N=54)	1.Depression: PHQ-9<br>2.Stress: SPICC	Postintervention<br>6 months
(Shata, et al., 2017) [27]	Egypt	Informal caregivers<br>Int: 49.4, 61.8% female<br>Con: 48.0, 69.5% female	Group psychoeducation (two sessions) +brief group CBT (six sessions, skills of handling problematic<br>Behaviors +skill of coping and stress management) +group support sessions<br>2 months (N=55)	Black control (N=59)	1.Depression: HDRS<br>2. Anxiety: TMAS<br>3.Burdem: ZBI	Postintervention<br>5 months
(Waldorff, et al., 2012) [28]	Denmark	Informal caregivers	(Dyadic)<br>Counselling sessions +course (basic knowledge +experiences and coping strategies)	Usual care (N=143)	1.Depression: GDS	Postintervention

			+additional intervention-telephone follow-up<br>+local support<br>8-12 months (N=128)			
(Yao, 2011) [29]	China	Family caregivers<br>Mean age: 64.2, 50.6% female	Cognitive behavior intervention (Group discussion, situational training, role playing) + transitional care<br>6 months (N=44)	Usual care (N=45)	1. Burden: CBI<br>2.Positive aspects of caregiving	Postintervention
(Yoo, et al., 2019) [30]	Korea	Family caregivers<br>Int: 65.9, 73.7% female<br>Con: 63.3, 84.2% female	Education +psychological support (CBT) +daily memory training +stress coping<br>8-10 weeks (N=19)	Wait-list control group (N=19)	1.Burden：ZBI<br>2.Depression ：GDS<br>3.Well-being: PGCMS<br>4.Positive effect: PANAS	Postintervention

(Zhu, et al., 2016) [31]	China	Family caregivers<br>Int: 57.4, 64.5% female<br>Con: 59.2, 71.7% female	Collaborative care intervention:<br>Social support (Wechat) +knowledge education + care skills instruction + psychology guidance<br>4 months (N=62)	Usual care (N=60)	1.Burden: CBI	Postintervention

Note: Int = Intervention group; Con = Control group; * Psychoeducation: A single-component intervention (a structured approach to providing information [32]); CES-D = Centre for Epidemiologic Studies Depression Scale; RSCSE = Revised Scale for Caregiving Self-Efficacy; RAS = The seven-item relationship assessment scale; REACH = The Resources for Enhancing Alzheimer's Caregiver Health; ZBI = the Zarit caregiver burden interview; PHQ = the Patient Health Questionnaire; HADS-A = The Hospital Anxiety and Depression Scale; FCBI = Family Caregiving Burden Inventory; PSS-14 = the 14-item Perceived Stress Scale; WHOQoL-BREF = The World Health Organization Quality of Life Measure-Brief Version; BDI-II = The second version of the Beck Depression Inventory; NHP = Nottingham Health Profile; VAS = visual analog scales; MT4C = My Tools 4 Care; GSES = The General Self-Efficacy Scale; SCL-90 = Symptom Checklist 90; The COPE program = The Care of Persons with Dementia in their Environments program; CarerQol-VAS = Care-related Quality of Life instrument- visual analog scales; IRI = The Interpersonal Reactivity Index; SSCQ = Sense of competence, the Short Sense of Competence Questionnaire; SDS = Self-rating depression scale; SAS = self-rating anxiety scale; BRS = Brief Resilience Scale; SCQ = the Sense of Competence Questionnaire; BSFC = Burden Scale for Family Caregivers; SPICC = The Dutch Self-Perceived Pressure from family care; HDRS = Arabic version of the Hamilton Depression Rating Scale; TMAS = the Taylor Manifest Anxiety Scale; GDS = Geriatric depression scale; PGCMS = The Korean version of the Philadelphia Geriatric Center Morale Scale; PANAS = The Positive Affect and Negative Affect Schedule.

References

1. Au, A.; Wong, M.K.; Leung, L.M.; Leung, P.; Wong, A. Telephone-assisted pleasant-event scheduling to enhance well-being of caregivers of people with dementia: a randomised controlled trial. Hong Kong Med. J. 2014, 20, 30-33.
2. Au, A.; Gallagher-Thompson, D.; Wong, M.K.; Leung, J.; Chan, W.C.; Chan, C.C.; Lu, H.J.; Lai, M.K.; Chan, K. Behavioral activation for dementia caregivers: scheduling pleasant events and enhancing communications. Clin. Interv. Aging 2015, 10, 611-619, doi:10.2147/cia.s72348.
3. Belle, S.H.; Burgio, L.; Burns, R.; Coon, D.; Czaja, S.J.; Gallagher-Thompson, D.; Gitlin, L.N.; Klinger, J.; Koepke, K.M.; Lee, C.C.; et al. Enhancing the quality of life of dementia caregivers from different ethnic or racial groups - A randomized, controlled trial. Ann. Intern. Med. 2006, 145, 727-738, doi:10.7326/0003-4819-145-10-200611210-00005.
4. Berwig, M.; Heinrich, S.; Spahlholz, J.; Hallensleben, N.; Brahler, E.; Gertz, H.-J. Individualized support for informal caregivers of people with dementia - effectiveness of the German adaptation of REACH II. BMC Geriatr. 2017, 17, 286, doi:https://dx.doi.org/10.1186/s12877-017-0678-y.
5. Birkenhäger-Gillesse, E.G.; Achterberg, W.P.; Janus, S.I.M.; Kollen, B.J.; Zuidema, S.U. Effects of caregiver dementia training in caregiver-patient dyads: A randomized controlled study. Int. J. Geriatr. Psychiatry 2020, 35, 1376-1384, doi:10.1002/gps.5378.
6. Blom, M.M.; Zarit, S.H.; Groot Zwaaftink, R.B.; Cuijpers, P.; Pot, A.M. Effectiveness of an Internet intervention for family caregivers of people with dementia: results of a randomized controlled trial. PLoS One 2015, 10, e0116622, doi:10.1371/journal.pone.0116622.
7. Chien, W.T.; Lee, I.Y.M. Randomized controlled trial of a dementia care programme for families of home-resided older people with dementia. J. Adv. Nurs. 2011, 67, 774-787, doi:10.1111/j.1365-2648.2010.05537.x.
8. Cristancho-Lacroix, V.; Wrobel, J.; Cantegreil-Kallen, I.; Dub, T.; Rouquette, A.; Rigaud, A.-S. A web-based psychoeducational program for informal caregivers of patients with Alzheimer's disease: a pilot randomized controlled trial. J. Med. Internet Res. 2015, 17, e117-e117, doi:10.2196/jmir.3717.
9. Duggleby, W.; Ploeg, J.; McAiney, C.; Peacock, S.; Fisher, K.; Ghosh, S.; Markle-Reid, M.; Swindle, J.; Williams, A.; Triscott, J.A.; et al. Web-Based intervention for family carers of persons with dementia and multiple chronic conditions (My Tools 4 Care): pragmatic randomized controlled trial. J. Med. Internet Res. 2018, 20, e10484, doi:https://dx.doi.org/10.2196/10484.
10. Elliott, A.F.; Burgio, L.D.; Decoster, J. Enhancing caregiver health: findings from the resources for enhancing Alzheimer's caregiver health II intervention. J. Am. Geriatr. Soc. 2010, 58, 30-37, doi:10.1111/j.1532-5415.2009.02631.x.
11. Fei, J.X.; Zhao, X.P.; Shen, L.Z. Investigation on nursing intervention improving coping style and emotional disorder of relatives of the senile dementia in hospital. Chinese Journal Of Modern Nursing 2009, 15, 2249-2251, doi:10.3760/cma.j.issn.1674-2907.2009.23.007. (in Chinese)
12. Gitlin, L.N.; Winter, L.; Dennis, M.P.; Hodgson, N.; Hauck, W.W. A biobehavioral home-based intervention and the well-being of patients with dementia and their caregivers: the COPE randomized trial. JAMA 2010, 304, 983-991, doi:10.1001/jama.2010.1253.
13. Gonyea, J.G.; O'Connor, M.K.; Boyle, P.A. Project CARE: a randomized controlled trial of a behavioral intervention group for Alzheimer's disease caregivers. The Gerontologist 2006, 46, 827-832.
14. Han, A.; Kim, T.H.; Hong, H. A factorial randomized controlled trial to examine separate and combined effects of a simulation-based empathy enhancement program and a lecture-based education program on family caregivers of people with dementia. Aging Ment. Health 2020, 1-11, doi:https://dx.doi.org/10.1080/13607863.2020.1768214.
15. Hattink, B.; Meiland, F.; van der Roest, H.; Kevern, P.; Abiuso, F.; Bengtsson, J.; Giuliano, A.; Duca, A.; Sanders, J.; Basnett, F.; et al. Web-based STAR E-Learning course increases empathy and understanding in dementia caregivers: results from a randomized controlled trial in the Netherlands and the United Kingdom. J. Med. Internet Res. 2015, 17, 15, doi:10.2196/jmir.4025.

16. Hepburn, K.W.; Tornatore, J.; Center, B.; Ostwald, S.W. Dementia family caregiver training: affecting beliefs about caregiving and caregiver outcomes. J. Am. Geriatr. Soc. 2001, 49, 450-457, doi:10.1046/j.1532-5415.2001.49090.x.
17. Huang, Y.N. Impact of comprehensive intervention on caregivers of patients with Alzheimer's disease. Health Guide: Medical research 2015, 0, 66-67. (in Chinese)
18. Kor, P.P.K.; Liu, J.Y.W.; Chien, W.T. Effects of a modified mindfulness-based cognitive therapy for family caregivers of people with dementia: A pilot randomized controlled trial. Int. J. Nurs. Stud. 2019, 98, 107-117, doi:https://doi.org/10.1016/j.ijnurstu.2019.02.020.
19. Laakkonen, M.-L.; Kautiainen, H.; Holtta, E.; Savikko, N.; Tilvis, R.S.; Strandberg, T.E.; Pitkala, K.H. Effects of self-management groups for people with dementia and their spouses--randomized controlled trial. J. Am. Geriatr. Soc. 2016, 64, 752-760, doi:https://dx.doi.org/10.1111/jgs.14055.
20. Liu, Q.S.; Shang, S.M.; Wang, Z.W.; Fu, Y.; Yue, P.; Zhang, H.; Liu, Y. Effects of a one-year nursing intervention on depression and anxiety in family caregivers of homebound patients with dementia. Chinese Journal Of Behavioral Medical Science 2007, 16, 172-174, doi:10.3760/cma.j.issn.1674-6554.2007.02.035. (in Chinese)
21. Luo, L.C.; Wang, J.A. Effect evaluation of nursing intervention on alleviating depression and anxiety among caregivers with Alzheimer's disease at home. Today Nurse 2012, 0, 22-24. (in Chinese)
22. Marriott, A.; Donaldson, C.; Tarrier, N.; Burns, A. Effectiveness of cognitive-behavioural family intervention in reducing the burden of care in carers of patients with Alzheimer's disease. Br. J. Psychiatry 2000, 176, 557-562, doi:10.1192/bjp.176.6.557.
23. Metcalfe, A.; Jones, B.; Mayer, J.; Gage, H.; Oyebode, J.; Boucault, S.; Aloui, S.; Schwertel, U.; Böhm, M.; Tezenas du Montcel, S.; et al. Online information and support for carers of people with young-onset dementia: A multi-site randomised controlled pilot study. Int. J. Geriatr. Psychiatry 2019, 34, 1455-1464, doi:10.1002/gps.5154.
24. Pan, Y.X.; Zhang, H.Y. Study of the effect of psychosocial intervention on depressive psychology and care burden of family caregivers of dementia patients. Shanghai Medical & Pharmaceutical Journal 2020, 41, 13-15,49. (in Chinese)
25. Possin, K.L.; Merrilees, J.J.; Dulaney, S.; Bonasera, S.J.; Chiong, W.; Lee, K.; Hooper, S.M.; Allen, I.E.; Braley, T.; Bernstein, A.; et al. Effect of collaborative dementia care via telephone and internet on quality of life, caregiver well-being, and health care use: The Care Ecosystem randomized clinical trial. JAMA Internal Medicine 2019, 179, 1658-1667, doi:10.1001/jamainternmed.2019.4101.
26. Prick, A.-E.; de Lange, J.; Twisk, J.; Pot, A.M. The effects of a multi-component dyadic intervention on the psychological distress of family caregivers providing care to people with dementia: a randomized controlled trial. Int. Psychogeriatr. 2015, 27, 2031-2044, doi:https://dx.doi.org/10.1017/S104161021500071X.
27. Shata, Z.N.; Amin, M.R.; El-Kady, H.M.; Abu-Nazel, M.W. Efficacy of a multi-component psychosocial intervention program for caregivers of persons living with neurocognitive disorders, Alexandria, Egypt: A randomized controlled trial. Avicenna journal of medicine 2017, 7, 54-63, doi:10.4103/2231-0770.203610.
28. Waldorff, F.B.; Buss, D.V.; Eckermann, A.; Rasmussen, M.L.H.; Keiding, N.; Rishøj, S.; Siersma, V.; Sørensen, J.; Sørensen, L.V.; Vogel, A.; et al. Efficacy of psychosocial intervention in patients with mild Alzheimer's disease: the multicentre, rater blinded, randomised Danish Alzheimer Intervention Study (DAISY). BMJ : British Medical Journal 2012, 345, e4693, doi:10.1136/bmj.e4693.
29. Yao, A.H. Effect of cognitive-behavior intervention on caregivers of alzheimer's disease patients. Chinese Journal of Modern Nursing 2011, 17, 373-375, doi:10.3760/cma.j.issn.1674-2907.2011.04.001. (in Chinese)
30. Yoo, R.; Yeom, J.; Kim, G.H.; Park, H.K.; Kang, Y.; Hwang, J.; Choi, S.H.; Na, H.R.; Cho, S.J.; Yu, K.H.; et al. A multicenter, randomized clinical trial to assess the efficacy of a therapeutic intervention program for caregivers of people with dementia. J. Clin. Neurol. 2019, 15, 235-242, doi:https://dx.doi.org/10.3988/jcn.2019.15.2.235.
31. Zhu, B.; Lin, Z.; He, X.J.; Shao, Z.M. Influence of collaborative care model on the burden of family caregivers of senile dementia patients in the community. Modern Medical Journal 2016, 44, 1780-1784, doi:10.3969/j.issn.1671-7562.2016.12.034. (in Chinese)
32. Pink, J.; O'Brien, J.; Robinson, L.; Longson, D. Dementia: Assessment, Management and Support: Summary of Updated NICE Guidance. BMJ **2018**, *361*, k2438, doi:10.1136/bmj.k2438.
