# Supplementary material for: The Effectiveness of Multi-Component Interventions on the Positive and Negative Aspects of Well-Being among Informal Caregivers of People with Dementia: A Systematic Review and Meta-Analysis
Source: Int J Environ Res Public Health. 2022 Jun 7;19(12):6973. doi: 10.3390/ijerph19126973 (PMC9222573; doi:10.3390/ijerph19126973)
Supplement: Supplementary file 1 [file ijerph-19-06973-s001.zip › Table S4. The results of subgroup analysis by delivery methods and participant types.pdf]

Table S4. The results of subgroup analysis by delivery methods and participant types.

Outcomes		Total effects size P [SMD (95%CI)]	Delivery methods P [SMD (95%CI)]				Participants P [SMD (95%CI)]		
			Subgroup Differences (P)	Face-to-face	Telephone	Online	Subgroup Differences (P)	Only caregivers	dyadic
**Primary outcomes**	Subjective well-being	<0.001*<br>[0.41(0.28, 0.54)]	0.12	<0.001<br>[0.53(0.33, 0.73)]	0.0001<br>[0.35(0.17, 0.53)]	0.94<br>[-0.02(-0.58, 0.54)]	0.16	<0.001<br>[0.57(0.31, 0.82)]	<0.001<br>[0.35(0.20, 0.50)]
**Secondary outcomes**	Depression	0.001*<br>[-0.29(-0.46, -0.11)]	0.46	0.02<br>[-0.35(-0.65, -0.06)]	0.02<br>[-0.39(-0.75, -0.03)]	0.04<br>[-0.19(-0.35, -0.02)]	0.04*	<0.001<br>[-0.42(-0.62, -0.22)]	0.42<br>[-0.09(-0.32, 0.14)]
	Burden	0.0003*<br>[-0.34(-0.53, -0.16)]	0.02*	<0.001<br>[-0.50(-0.76, -0.25)]	-	0.07<br>[-0.15(-0.31, 0.01)]	0.05	<0.001<br>[-0.42(-0.64, -0.19)]	0.05<br>[-0.14(-0.28, 0.00)]
	Anxiety	<0.001*<br>[-0.53(-0.78, -0.27)]	0.35	0.0003<br>[-0.57(-0.87, -0.26)]	-	0.02<br>[-0.36(-0.66, -0.07)]	-	-	-
	Stress	0.06<br>[-0.23(-0.47, 0.01)]	0.99	0.15<br>[-0.23(-0.54, 0.08)]	-	0.23<br>[-0.23(-0.61, 0.14)]	0.95	0.15<br>[-0.24(-0.59, 0.09)]	0.22<br>[-0.22(-0.58, 0.14)]

Note: SMD=standard mean difference; CI=confidential interval; *P<0.05.
