# Supplementary figures and images for: The Effectiveness of Multi-Component Interventions on the Positive and Negative Aspects of Well-Being among Informal Caregivers of People with Dementia: A Systematic Review and Meta-Analysis
Source: Int J Environ Res Public Health. 2022 Jun 7;19(12):6973. doi: 10.3390/ijerph19126973 (PMC9222573; doi:10.3390/ijerph19126973)

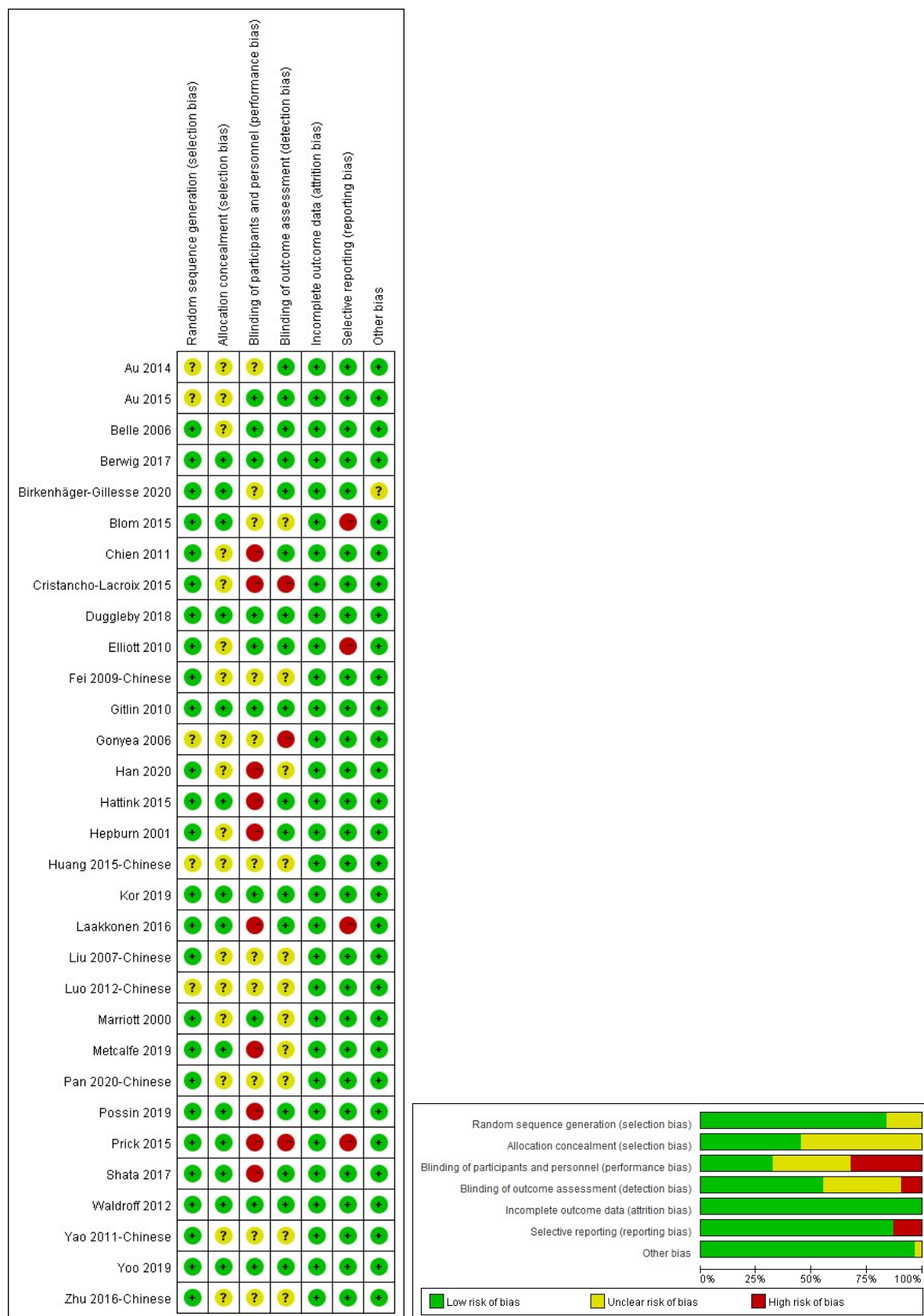

**Figure S1.** Risk of bias in included studies.

Supplement: Supplementary file 1 [file ijerph-19-06973-s001.zip › Figure S1_Risk of bias in included studies.pdf]
